# Effect of Hotel Air Quality Management on Guests’ Cognitive and Affective Images and Revisit Intentions

**DOI:** 10.3390/ijerph18179346

**Published:** 2021-09-04

**Authors:** Junghyun Park, Jae Leame Yoo, Jongsik Yu

**Affiliations:** 1 College of Hospitality and Tourism Management, Sejong University, 98 Gunja-Dong, Gwanjin-gu, Seoul 143747, Korea; junghyun.peterpark@gmail.com; 2 Devision of Aeronautics, Cheongju University, 298 Daesung-ro, Cheongwon-gu, Cheongju-si 28503, Korea; jlyoo@cju.ac.kr; 3 College of Business Division of Tourism and Hotel Management, Cheongju University, 298 Daesung-ro, Cheongwon-gu, Cheongju-si 28503, Korea

**Keywords:** air pollution control devices, air quality monitoring system, cognitive image, affective image, revisit intentions

## Abstract

Although city air pollution levels significantly affect the hotel industry, few studies have addressed the impact of air quality management on guests’ cognitive and affective image formation and revisit intentions. Therefore, this research examined the effects of hotel air quality management on the formation of guests’ cognitive and affective images and their revisit intentions. A total of 322 valid samples were obtained by surveying hotel guests who had perceived hotel air quality management activities in the past year, with SPSS 22.0 (IBM, New York, NY, USA) and AMOS 22.0 (IBM, New York, NY, USA) employed for the empirical analysis. The cognitive and affective image constructs revealed that cognitive (perceived value and perceived quality) image influenced revisit intentions but affective image did not. These results provide insights into the need for hotel managers to develop positive cognitive and emotional images through good air quality management and the need to induce customers to revisit based on these images.

## 1. Introduction

Industrialization has brought many advantages, such as improved medical care, productivity, time conservation, and better living standards. However, it has also resulted in negative environmental consequences, such as water shortages, solid waste pollution, air pollution, and land pollution [1]. Particularly, air quality has become a serious city problem that endangers the health and quality of life of inhabitants and causes many disease-related heart and lung problems, with the World Health Organization claiming that an estimated seven million people worldwide die each year from air pollution-related causes [2]. Air pollution is usually thought of as emissions from factories and car exhausts. However, numerous indoor air pollutants, from burning substances, naturally occurring radon gas, and some construction materials have also been recognized as significant risk factors [3]. Therefore, good indoor air quality management, particularly in schools, multistory offices, and hospitality service providers can protect human health [4].

Air quality standards can influence visitor perceptions and experiences in tourist areas and the hotel sector. Visible air pollutants, such as smoke and dust, have an obvious impact on stay quality as poor air quality can obstruct views and degrade sensory inputs [5,6]. However, invisible air pollutants, such as carbon monoxide, nitrogen dioxide, and sulfur dioxide, are unlikely to result in similar perceptions and feelings [6]. The hotel industry needs to have high standards to ensure good overall customer experiences [7]. Particularly, hotel guests expect clean, fresh air during their stay, the absence of which could affect their desire to return to specific hotel brands [8,9]. Therefore, maintaining good hotel air quality and minimizing pollution require effective air control and monitoring systems to adequately filter and clean the air.

Air quality management is essential to the hotel industry, as poor indoor air can harm staff, guests, and customers. Although there have been many studies on air quality and consumer behavior [7,8,9,10], there has been less research on the complex relationship between a hotel’s indoor air quality management and consumer image perceptions. Considering that bad air quality can have a detrimental effect on human health and cause major diseases, it is necessary to verify the relationship between air quality in hotels and consumer behavior. Therefore, to expand existing research on hotel guest intentions, this study examined the effect of hotel air quality management on customers’ cognitive and affective images. To achieve the aims of the study, the properties of air quality management were identified through a qualitative approach. Additionally, the relationships between the properties of air quality management—which were identified through a quantitative approach—and the hotel image as well as revisit intentions were investigated. To be more specific, the purpose of the study was: (1) to identify the properties of air quality management through a qualitative approach using a literature review and focus group interviews; (2) to verify the relationship between the properties of air quality management and the hotel’s cognitive and affective images; and (3) to verify the relationship between revisit intentions and the hotel’s cognitive and affective images. The results of this study are expected to provide very meaningful academic implications for studies on the hotel industry, and furthermore, provide significant strategic implications for hotel practitioners.

## 2. Literature Review

### 2.1. Air Quality Management Devices and Monitoring System

Air pollution results from fossil fuel power generation, heavy manufacturing, and chemical industry production [11]. Air pollution has an adverse and direct impact on human health. Specifically, it is known to cause a variety of different diseases and even result in death [12]. In other words, factors that negatively affect air quality, such as air pollution, can cause serious problems in human life. Particularly, poor hotel air quality air can influence staff, guests, and the customers who visit hotel establishments [10,13]. However, hotels have a responsibility to ensure the hotel air quality environment is safe, especially when hotel guests usually spend at least one-third of their travel time in their rooms eating, working, sleeping, or exercising [13]. This means that proper ventilation, boiler and heating system maintenance, and integrated air quality solutions are needed to mitigate potential airborne contaminants. Chen and Hildemann [14] found unhealthy air in the occupied rooms of a sample of hotels in a large industrial district in China where the outdoor air quality was poor and claimed that the primary pollution sources were indoors rather than the ambient environment of the industrial region [15]. As the airtight and energy-efficient design of modern hotels, houses, and buildings can result in indoor air pollution levels higher than the outdoor environment, indoor air quality management is vital, especially in the hotel industry [15,16,17].

In the highly competitive hotel industry, hotels must provide multifaceted comfort and services to guests and an ambient internal environment, which requires air quality, temperature, odor, light, and sound controls [18,19]. Indoor hotel air quality maintenance requires high-quality air purification devices to filter the air and remove harmful pollutants that can contaminate the indoor environment. Some of the most common hotel air quality solutions have been HVAC filters and stand-alone air purifiers [13] as well as quality monitoring devices to detect common pollutants, such as carbon dioxide, dust and particles, formaldehyde, and odors. Air quality monitoring systems, such as air-gas sensors, scrubbers, cyclones, mist collectors, biofilters, and incinerators are used to regulate and remove potentially harmful pollutants in hotels, with these internal physical devices and monitoring systems being instrumental to guests’ images of a hotel [19]. A study found that guest images were composed of three sequential concepts: (a) cognitive, (b) affective, and (c) overall attitudinal concepts [20,21].

The cognitive image affects a guest’s emotional responses and is related to their beliefs and knowledge about the physical environment, that is, the “perceived value” and “perceived quality” [22]. The perceived value in the hotel industry comprises rest, room conditions, air quality, and atmosphere, and the perceived quality comprises the accommodation, value for money, service/staff quality, and the cleanliness of the environment [23,24]. Oltra and Sala [23] found that air quality could influence guests’ perceptions of hotel environments and impact cognitive and affective images and revisit intentions [24,25]. Therefore, on the basis of previous studies, this study proposed the following hypotheses.

**Hypothesis 1 (H1).**  *Air pollution control devices have a positive effect on perceived value.*

**Hypothesis 2 (H2).**  *Air pollution control devices have a positive effect on perceived quality.*

**Hypothesis 3 (H3).**  *Air pollution control devices have a positive effect on affective image.*

**Hypothesis 4 (H4).**  *Air quality monitoring systems have a positive effect on perceived value.*

**Hypothesis 5 (H5).**  *Air quality monitoring systems have a positive effect on perceived quality.*

**Hypothesis 6 (H6).**  *Air quality monitoring systems have a positive effect on the affective image.*

### 2.2. Cognitive and Affective Image

An image has been defined as “the sum total of impressions the consumer receives from many sources” [26] and can also define an individual’s or group’s beliefs, behaviors, or impressions regarding a subject. Hotel industry physical environment studies have found that guest satisfaction is influenced by their specific image of the hotel [27] and the physical hotel environment [28,29,30]. Lee, Hsu, Han, and Kim [31] concluded that the cognitive and affective images of hotels were sub-dimensions of the overall image and claimed that guest thought development proceeds through both cognitive and affective images. Recent studies have also confirmed that satisfaction with a hotel’s image (cognitive and affective) was an antecedent to revisit intentions and word of mouth recommendations [32].

Martin and Bosque [22] defined the cognitive image as an individual’s knowledge and beliefs about a destination, that is, the destination image is the tourist’s evaluation of its value and quality based on their perceptual–cognitive evaluation of the destination’s objective or physical attributes [33]. To define cognitive images, Baloglu and McCleary [34] used three cognitive dimensions: experience quality, attractions, and value/environment. For tourists, the cognitive and affective phases form the overall tourist image conception [35] and comprise functional and psychological facets of perceived quality and value [36], with perceived quality being the overall perception of performance superiority or inferiority and perceived value being the comprehensive evaluation of product/service utility based on the perception of what has been obtained and given [32,37]. Milfelner et al. [38] found that perceived hotel quality and value had a positive relationship and that both attributes strongly impacted hotel guest satisfaction and revisit intentions.

Affectivity is another critical factor in a guest’s favorable image formation. Baloglu et al. [34] claimed that the evaluation of favorable, negative, or neutral feelings toward a location’s characteristics was related to the destination’s affective image components. Russell, Ward, and Pratt [35] measured the affective image on four dimensions: “pleasant/unpleasant”, “relaxing/distressing”, “arousing/sleepy”, and “exciting/gloomy”, all of which affected the tourist’s emotional image of the destination and motivated them to revisit the same place. Therefore, positive cognitive and affective factors can increase a hotel guest’s revisit intentions even if their motivation is not strong [31,39,40], that is, the affective image plays a crucial role in triggering the guest’s overall image and revisit intentions. On the basis of these considerations, the following hypotheses were proposed.

**Hypothesis 7 (H7).**  *Cognitive image (perceived quality) has a positive effect on revisit intentions.*

**Hypothesis 8 (H8).**  *Cognitive image (perceived value) has a positive effect on revisit intentions.*

**Hypothesis 9 (H9).**  *Affective image has a positive effect on revisit intentions.*

### 2.3. Revisit Intentions

In the midst of ongoing competition in the service industry, enhancing customer retention and revisit intentions plays a key role in the survival and long-term success of a company [41]. Having customers revisit saves time and money, and is thus more effective than creating new customers [42]. Revisit intentions are the intentions to return to or revisit a particular place. Warshaw and Davis [43] defined revisit intentions as “the degree to which a person has formulated conscious plans to perform or not perform some specified future behavior”. In the hotel industry, many studies have found that maximizing revenue, growth, and performance significantly affects revisit intentions [19,29,31,44,45] and is also related to numerous variables, such as satisfaction, image, motivation, and quality. When guests have a positive hotel experience, they tend to be more satisfied with the hotel, which increases their desire to return. Particularly, many studies have found a causal relationship between positive image and revisit intentions. For example, Lee et al. [31] found that the green hotel’s overall cognitive and affective images positively affected revisit intentions and the formation of hotel guest loyalty, and Han, Hsu, and Lee [46] concluded that overall image was a critical driving factor in a guest’s revisit intentions. Therefore, a guest’s revisit intentions, that is, their cognitive and affective images, are an essential factor in assessing hotel industry performance.

## 3. Methods

### 3.1. Qualitative Approach

This study attempted to explore specific properties of air quality management to emphasize its importance in hotels, using a qualitative approach. This was in line with the current situation where the importance of air quality is being emphasized worldwide. To determine the properties of air quality management, the existing literature was reviewed and focus group interviews were conducted, following Han et al. [36] and Maxwell [47]. For the literature review, papers, articles, government publications, and research materials related to air quality were analyzed. The focus groups included university professors who specialize in hotel and tourism fields, and hotel staff. Further, various materials (e.g., papers, articles, and research materials) related to the subject of this study were provided to the respondents before conducting the focus group interview; the focus group members were asked to carefully review them. The members of the focus group were provided with materials that had already been published by prominent journals and institutions following various verification processes. Moreover, since these experts had voluntarily agreed to participate in this study and agreed with its purpose, it can be said that the materials provided in this study were thoroughly reviewed. The purpose was to improve the quality of the focus group interviews and broaden its members’ personal thoughts and ideas. Through the interviews, information regarding the members’ varied opinions, knowledge, and ideas on the subject of this study were obtained, which was further shared with other members of the focus group. Discussions were held to enhance each focus group member’s opinions, knowledge, and ideas and resolve the differences in their opinions. Through this process, a list of properties of air quality management was prepared, which were agreed on by all the members. In this study, a total of 11 attributes were explored through a literature review and focus group interviews, out of which three attributes were excluded because they were considered to be inconsistent with the subject of this study or did not convey meaning accurately. Therefore, a total of eight attributes were derived in this study, which were classified into the two categories of air pollution control devices and air quality monitoring systems, using the qualitative approach proposed by Spiggle [48].

### 3.2. Measurement Tools for Other Constructs

Based on previous studies, this study used measurement items with proven validity and reliability to measure perceived value, perceived quality, affective image, and revisit intentions, excluding air quality management that was derived through a qualitative approach. Specifically, three questions were used to measure perceived value and perceived quality, based on Varki and Colgate [49]. In addition, four questions were used to measure affective image, following Lee et al. [31]. Finally, based on Hennig-Thurau et al. [50], three questions were used to measure revisit intentions. All the questionnaire items used in this study were multi-items, and a seven-point Likert scale ranging from 1 (strongly disagree) to 7 (strongly agree) was used to analyze them. In addition, a pre-test was conducted so that the survey respondents could understand the contents of the questionnaire more clearly, such that the contents of the questionnaire could be revised and supplemented. The pre-test was conducted on university professors and graduate students specializing in hotel and tourism management, and hotel staff.

### 3.3. Data Collection and Sample Characteristics

In this study, the data used in the empirical analysis were collected using the web-based system of an internet market research firm. In the questionnaire, the purpose of the study was clearly explained, and it was stated that the confidentiality of the respondents would be ensured. The survey respondents, limited to customers who had used a hotel during the past year, were randomly selected through a screening question sent via e-mail using the web-based system. In the screening question, first-class hotels located in the capital and big cities of Korea were presented as examples, and respondents were asked to select one or more of the hotels provided. Accordingly, a total of 326 observations were obtained, out of which four observations were excluded from the study because they did not provide an unbiased response. Therefore, the empirical analysis in this study was finally performed using 322 observations. The characteristics of the sample were as follows. First, it was confirmed 153 men (47.5%) and 169 women (52.5%) participated in the interviews. Second, as for the age of the respondents, it was confirmed that 69, 122, 108, and 23 of them were in their 20s (21.4%), 30s (37.9%), 40s (33.5%), and 50s or older (7.1%), respectively. Third, as for the educational background of the respondents, it was found that nine of them were high school graduates (2.8%), 45 of them were junior college graduates (14%), 215 of them were university degree holders (66.8%), and 53 of them were advanced degree holders (16.5%). Finally, in the context of the annual income earned by the respondents, it was found that 97 (30.1%) of them had an annual income of less than $40,000, 174 (54%) of them had an annual income of more than $40,000 but less than $70,000, and 51 (15.8%) of them had an annual income of more than $70,000.

## 4. Results

### 4.1. Exploratory Factor Analysis

In this study, exploratory factor analysis (EFA) was performed to determine the properties of air quality management. The varimax orthogonal rotation method using principal component analysis was used to conduct the EFA. From the results of the EFA, it could be observed that the Kaiser–Meyer–Olkin value was 0.876 and the Bartlett value was statistically significant (*p* < 0.001). Based on these results, it was possible to say that the selection of variables was appropriate in this study. In addition, two factors with an eigenvalue of 1 or more were derived, such that the total variance was found to be 70.232%. Out of the two derived factors, the first factor was “air pollution control devices”, which consisted of a total of four items; its variance was found to be 53.257%. The second factor was “air quality monitoring systems”, which consisted of a total of four items; its variance was found to be 16.975%. Next, a reliability analysis was performed to verify internal consistency. As a result, the Cronbach’s alpha was 0.878 for “air pollution control devices” (the first factor) and 0.900 for “air quality monitoring systems” (the second factor). According to Hair et al. [51], there exists no problem in terms of internal consistency for values that are equal to or greater than 0.7. Therefore, it was concluded that there was no problem with the internal consistency of either factor used in this study, since their values were higher than 0.7. In addition, since the factor loadings of the measurement items presented in this study varied between 0.788 and 0.885, it was possible to say that both attributes derived in this study were statistically significant. The detailed results of the EFA are shown in Table 1.

### 4.2. Results of the Measurement Model

In this study, confirmatory factor analysis (CFA) was performed to confirm the unidimensionality of the scale and the validity and reliability of the measurement model. As per the CFA results pertaining to the fit of the measurement model, it was observed that χ^2^ = 351.370, *df* = 155, *p* < 0.001, χ^2^/*df* = 2.267, RMSEA = 0.063, CFI = 0.937, and TLI = 0.923, indicating a statistically acceptable model fit. Next, the standardized regression weights were evaluated to confirm the reliability of the measurement items presented in this study; all the measurement items ranged from 0.581 to 0.874, which exceeded the criterion for a standardized regression weight of 0.5, confirming that all the presented measurement items were reliable. According to Fornell and Larcker [52], there is no problem with the internal consistency and convergent validity of measurement variables if the average variance extracted (AVE) value is 0.5 or more, and the composite reliability (CR) value is 0.7 or more. Therefore, the AVE and CR values were checked to confirm the internal consistency and convergent validity of the measurement variables; the AVE values were observed to range from 0.600 to 0.644, while the CR values ranged from 0.817 to 0.878. Therefore, it was possible to say that there was no problem with the internal consistency and convergent validity of the measurement variables presented in this study. Finally, tests for discriminant validity were conducted to verify the differentiation in the constructs presented in this study. It can be said that there is no problem in the context of discriminant validity when the AVE value is larger than the squared value of the correlation coefficient of the latent variables [52]. The discriminant validity analysis performed in this study showed that the AVE value was greater than the squared value of the correlation coefficient of the variables. Therefore, the discriminant validity among the variables presented in this study was found to be secure. The detailed results of the CFA are shown in Table 2.

### 4.3. Structural Equation Modeling

In this study, structural equation modeling was performed to test the hypotheses and the proposed conceptual framework. The results of the analysis were as follows. First, it was observed that χ^2^ = 397.937, *df* = 160, *p* < 0.001, χ^2^/*df* = 2.487, RMSEA = 0.068, CFI = 0.924, and TLI = 0.909, indicating a statistically acceptable model fit. Second, the nine hypotheses presented in this study were tested; the results are as follows. “Air pollution control devices” was found to have a statistically significant effect on perceived value (β = 0.271), perceived quality (β = 0.347), and affective image (β = 0.327). “Air quality monitoring systems” was also found to have a statistically significant effect on perceived value (β = 0.536), perceived quality (β = 0.580), and affective image (β = 0.562). Therefore, Hypotheses 1, 2, 3, 4, 5, and 6 were all accepted. Third, to verify Hypotheses 7, 8, and 9, the effect of the relationships between perceived value, perceived quality, and affective image on revisit intentions were verified. Accordingly, perceived value (β = 0.229) and perceived quality (β = 0.493) were found to have a statistically significant effect. However, affective image (β = 0.187) did not have a statistically significant effect on revisit intentions. Therefore, Hypotheses 7 and 8 were accepted but Hypothesis 9 was rejected.

**Figure 1:**
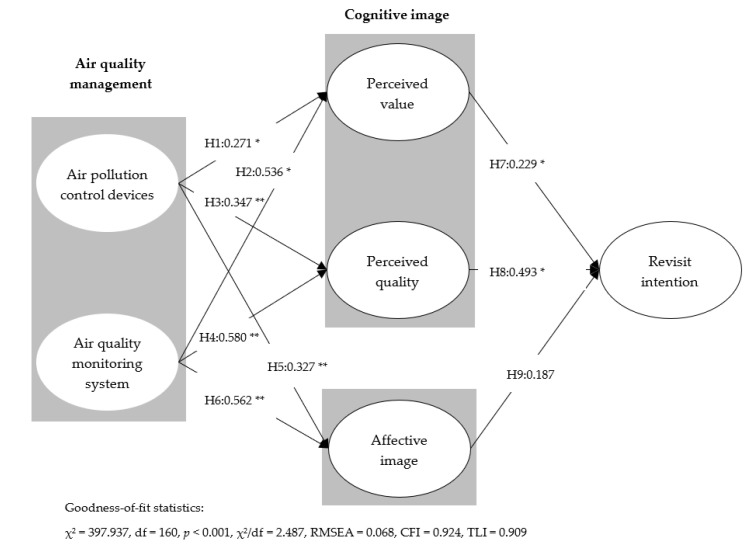
Structural model results and hypotheses testing. * *p* < 0.05, ** *p* < 0.01. Note. Goodness-of-fit statistics for the structural model: χ^2^ = 397.937, *df* = 160, *p* < 0.001, χ^2^/*df* = 2.487, RMSEA = 0.068, CFI = 0.924, TLI = 0.909.

According to Han and Ryu [53], it is very desirable to utilize the mediating framework within the theoretical model to understand the complex relationships of the research structure. Therefore, in this study, the indirect effect was verified using bootstrapping, based on Han and Ryu [53], in order to help understand the proposed research structure. By verifying the indirect effect, it was found that both the attributes of air quality management, that is, air pollution control devices (β = 0.294) and air quality monitoring systems (β = 0.514), presented in this study had statistically significant indirect effects on revisit intentions. Therefore, within the theoretical framework presented in this study, perceived value, perceived quality, and affective image were all proven to play a statistically significant mediating role. The detailed results are shown in Figure 1 and Table 3.

## 5. Discussion and Implications

Due to the changing industrial environment, there has been an increased public interest in environmental pollution, especially perceptions of air quality. As most hotel guests spend a great deal of time using hotel facilities, a hotel’s response to air quality significantly affects guests’ image perception. However, although air quality management is of critical importance, little hotel industry research has been conducted on air quality management and guests’ cognitive and affective images or their revisit intentions. Therefore, this study filled this research gap by investigating the influence of hotel air quality control devices and monitoring systems on guests’ cognitive and affective images and their revisit intentions. Specifically, the cognitive image was divided into perceived value and perceived quality, with the effects of these two variables on revisit intentions investigated on the basis of the affective image. A survey was conducted on 322 cases, the quantitative data from which were subjected to exploratory factor and empirical analyses, structural equation modeling, and an invariance test. It was found that all measurement items in the measurement model had appropriate levels of reliability and validity.

The conceptual framework presented in this study revealed that hotel air quality management affected guests’ cognitive and affective images of the hotel. The conceptual framework found that perceived value and perceived quality also explained guests’ revisit intentions. However, the relationship between affective image and revisit intentions was not found to be well explained in this study. The mediating role of the cognitive (perceived value and perceived quality) and affective images between air quality control devices and monitoring systems and revisit intentions was also examined. In summary, this study confirmed that the hotel industry’s air quality management systems positively affected the hotels’ cognitive image (perceived value, perceived quality) and that the cognitive image (perceived value, perceived quality) influenced guests’ revisit intentions, which suggested that diverse and specific hotel air quality management systems are needed.

On the basis of the results, this study presented nine hypotheses and provided several theoretical implications for hotel air quality management. First, this research revealed that the air quality control device and monitoring system sub-factors contributed to a positive image of a hotel and had a significant effect on perceived value, perceived quality, and affective images. As hotels have high daily human and machine activities, air quality can be significantly affected; therefore, stringent air quality control procedures are needed. The results of this study indicated that positive hotel value, quality perceptions, and affective images can be formed by maintaining good hotel air quality [10,13,16,17,24]. Hartmann, Ibáñez, and Sainz [54] also found that an effective hotel green positioning strategy (e.g., air quality management) had cognitive and affective benefits. Consequently, based on this and previous studies, hotel managers should actively adopt air quality management devices, such as dust collectors, air environment plants, and real-time air quality monitoring systems [10,13,15,55]. In addition, hotel managers need to be more active in promoting air pollution control devices and air quality monitoring systems, and in investing in various pieces of equipment and methods for air quality management. They can build a more positive customer perception and image of the hotel through such efforts. This study makes an important theoretical contribution by shedding light on the links between hotel air quality management and guests’ cognitive and affective images.

Second, both perceived value and perceived quality were found to have a positive effect on revisit intentions. These results also supported previous studies that found that positive cognitive images toward hotel air quality management induced positive behaviors [24,31,37] and also supported findings that guests’ thoughts progress through cognitive images [31,56], with the cognitive image relating to a guest’s perception (perceived value and quality) of a particular object (such as air quality) based on their evaluation of the attributes. In other words, hotel guests’ perceived value and perceived quality are closely related to guests’ revisit intentions, which directly affect the hotel business. However, unlike previous studies [22,36,40], the affective image, which is related to the personal feelings someone has toward a particular object, was not found to affect revisit intentions. Perugini and Bagozzi [57] claimed that the affective image was related to an individual’s pleasant or unpleasant emotions resulting from an engagement with a particular behavior. In line with this, the results of this study indicated that personal emotions and feelings toward the air quality management in a hotel were not a key factor in revisit intentions. Therefore, based on the hypotheses tested in this study, the results indicated that hotel air quality management activities were necessary strategies to elevate guests’ perceptions of the hotel’s value and quality and revisit intentions but were not effective in elevating guests’ affective image.

The examination of the indirect effects of cognitive (perceived value, perceived quality) and affective images on air quality control devices and revisit intentions, and air quality monitoring systems and revisit intentions, confirmed that air quality management activities had a statistically indirect effect through perceived value, perceived quality, and affective image on revisit intentions. Consequently, unlike the indirect effect test, the structural model estimation of the relationship between affective image and revisit intentions did not significantly affect these factors. However, the indirect effect test showed that the affective image acted as an important variable in the perception of the hotel industry’s air quality management on the intentions to revisit.

In this study, new variables, such as air pollution devices and air quality monitoring systems, were presented as sub-factors of air quality management. In other words, we have developed new variables that did not exist before using a qualitative approach, and demonstrated the relationship between air quality management and customer behavior through a quantitative approach. This has great theoretical significance in that it suggests a differentiated approach and new direction from existing studies on air quality. This study expanded on existing research by proposing that air quality control devices and air quality monitoring systems should be specific hotel air quality management strategies. In other words, providing guests with clean environments using hotel air quality management can positively affect a hotel’s cognitive and affective images and improve hotel performance. This study extended existing hotel industry air quality studies as the results highlighted the importance of hotel air quality management strategies in the formation of a positive guest image of the hotel.

Despite the various meaningful findings, this study had several limitations. First, as this study only examined hotel industry air quality management activities, the results cannot be generalized to other industries. Therefore, future research could expand this research to multiple industries and countries. Second, the data used for the empirical analysis were limited to the perceptions of Korean guests and possibly cannot be applied to other cultures. Third, this study only verified the effect of hotel air quality management activities on cognitive and affective images. Therefore, in subsequent studies, a greater number of image factors must be included to expand the study scope.

## 6. Conclusions

Due to increased concerns about air quality, internal air quality in establishments such as hotels has also attracted increased attention. Thus, in this study, the air quality management hotel activities were classified into air quality control devices and air quality monitoring systems, and the effects of these strategies on cognitive (perceived value, perceived quality) and affective images and revisit intentions were investigated. The empirical analysis found that hotel air quality management had a positive (+) effect on perceived value, perceived quality, and affective image, and perceived value and quality had a positive (+) effect on revisit intentions, but the affective image had a negative (+) effect on revisit intentions; however, the indirect effect test found that hotel air quality management had a positive (+) effect on revisit intentions through perceived value, perceived quality, and affective image. Hence, the purpose of this study was successfully achieved, and some meaningful implications have been provided.

## Figures and Tables

**Table 1:** Exploratory factor analysis results.

Factors	% of Variance	Factor Loadings	Cronbach’s Alpha
Factor 1: Air pollution control devices	53.257		0.878
1. This hotel actively uses special equipment (e.g., dust collectors) to remove fine dust and particulates and to eliminate volatile organic compounds.		0.823	
2. This hotel actively uses special equipment (e.g., air environment plants) to remove odors and harmful gases.		0.875	
3. This hotel constantly measures and manages air pollutant emissions at the hotel with automatic measuring devices.		0.885	
4. This hotel has installed indoor air purifiers to purify the air and remove various bad odors.		0.788	
Factor 2: Air quality monitoring systems	16.975		0.900
1. This hotel has a monitoring and management system to reduce greenhouse gas emissions.		0.842	
2. This hotel has a monitoring and management system to save energy.		0.864	
3. This hotel has a monitoring and management system to minimize the emission of hazardous air pollutants.		0.852	
4. This hotel has a system for monitoring and managing the air quality of all spaces used by hotel guests (e.g., guest rooms, restaurants, gyms, etc.).		0.831	

Total variance explained: 70.232. KMO measure of sampling adequacy: 0.876. Bartlett’s test of sphericity (*p* < 0.01).

**Table 2:** Measurement model assessment and correlations.

	(1)	(2)	(3)	(4)	(5)	(6)
Air pollution control devices (1)	1.000					
Air quality monitoring systems (2)	0.624 ^a^ (0.389) ^b^	1.000				
Perceived value (3)	0.591 (0.349)	0.681 (0.463)	1.000			
Perceived quality (4)	0.698 (0.487)	0.545 (0.297)	0.614 (0.376)	1.000		
Affective image (5)	0.659 (0.434)	0.529 (0.279)	0.527 (0.277)	0.697 (0.485)	1.000	
Revisit intentions (6)	0.586 (0.343)	0.617 (0.380)	0.638 (0.407)	0.501 (0.251)	0.630 (0.396)	1.000
Mean	5.451	4.941	5.277	5.464	5.060	5.187
SD	0.867	0.994	0.850	0.790	0.786	0.627
CR	0.863	0.878	0.824	0.832	0.817	0.831
AVE	0.614	0.644	0.611	0.623	0.600	0.622

Note. Goodness-of-fit statistics for the measurement model: χ^2^ = 351.370, *df* = 155, *p* < 0.001, χ^2^/*df* = 2.267, RMSEA = 0.063, CFI = 0.937, TLI = 0.923. ^a^ Correlations between the variables are below the diagonal. ^b^ The squared correlations between the variables are within the parentheses.

**Table 3:** The structural model results.

Hypothesized Paths			Coefficients	t-Values
H1: Air pollution control devices	→	Perceived value	0.271	3.458 **
H2: Air quality monitoring systems	→	Perceived value	0.536	6.850 **
H3: Air pollution control devices	→	Perceived quality	0.347	4.485 **
H4: Air quality monitoring systems	→	Perceived quality	0.580	7.414 **
H5: Air pollution control devices	→	Affective image	0.327	3.832 **
H6: Air quality monitoring systems	→	Affective image	0.562	6.471 **
H7: Perceived value	→	Revisit intentions	0.229	2.820 **
H8: Perceived quality	→	Revisit intentions	0.493	4.483 **
H9: Affective image	→	Revisit intentions	0.187	1.869
Indirect effect: β _Air pollution control devices_ _→ perceived value & perceived quality & affective image_ _→ revisit intentions_ = 0.294 ** β _Air quality monitoring systems_ _→ perceived value & perceived quality & affective image_ _→ revisit intentions_ = 0.514 **			Explained variance: R^2^ (Perceived value) = 0.542 R^2^ (Perceived quality) = 0.707 R^2^ (Affective image) = 0.652 R^2^ (Revisit intentions) = 0.647	

## Data Availability

The dataset used in this research are available upon request from the corresponding author. The data are not publicly available due to restrictions i.e., privacy or ethical.
